# NEK2 induces autophagy‐mediated bortezomib resistance by stabilizing Beclin‐1 in multiple myeloma

**DOI:** 10.1002/1878-0261.12641

**Published:** 2020-01-29

**Authors:** Jiliang Xia, Yanjuan He, Bin Meng, Shilian Chen, Jingyu Zhang, Xuan Wu, Yinghong Zhu, Yi Shen, Xiangling Feng, Yongjun Guan, Chunmei Kuang, Jiaojiao Guo, Qian Lei, Yangbowen Wu, Gang An, Guancheng Li, Lugui Qiu, Fenghuang Zhan, Wen Zhou

**Affiliations:** ^1^ Department of Hematology Xiangya Hospital Central South University Changsha China; ^2^ Key Laboratory for Carcinogenesis and Invasion Chinese Ministry of Education Key Laboratory of Carcinogenesis Chinese Ministry of Health Cancer Research Institute School of Basic Medical Sciences Central South University Changsha China; ^3^ Department of Orthopaedic Surgery Second Xiangya Hospital Central South University Changsha China; ^4^ Department of Medicine Division of Hematology, Oncology and Blood and Marrow Transplantation Holden Comprehensive Cancer Center University of Iowa IA USA; ^5^ Xiangya School of Public Health Central South University Changsha China; ^6^ State Key Laboratory of Experimental Hematology Institute of Hematology & Blood Diseases Hospital Chinese Academy of Medical Science & Peking Union Medical College Tianjin China

**Keywords:** autophagy, Beclin‐1, multiple myeloma, NEK2, ubiquitination

## Abstract

NEK2 is associated with drug resistance in multiple cancers. Our previous studies indicated that high NEK2 confers inferior survival in multiple myeloma (MM); thus, a better understanding of the mechanisms by which NEK2 induces drug resistance in MM is required. In this study, we discovered that NEK2 enhances MM cell autophagy, and a combination of autophagy inhibitor chloroquine (CQ) and chemotherapeutic bortezomib (BTZ) significantly prevents NEK2‐induced drug resistance in MM cells. Interestingly, NEK2 was found to bind and stabilize Beclin‐1 protein but did not affect its mRNA expression and phosphorylation. Moreover, autophagy enhanced by NEK2 was significantly prevented by knockdown of Beclin‐1 in MM cells, suggesting that Beclin‐1 mediates NEK2‐induced autophagy. Further studies demonstrated that Beclin‐1 ubiquitination is decreased through NEK2 interaction with USP7. Importantly, knockdown of Beclin‐1 sensitized NEK2‐overexpressing MM cells to BTZ *in vitro* and *in vivo*. In conclusion, we identify a novel mechanism whereby autophagy is activated by the complex of NEK2/USP7/Beclin‐1 in MM cells. Targeting the autophagy signaling pathway may provide a promising therapeutic strategy to overcome NEK2‐induced drug resistance in MM.

## Graphical abstract

Autophagy is associated with bortezomib resistance in multiple myeloma (MM). Here, we show that NEK2 stabilizes Beclin‐1 through USP7‐mediated deubiquitination, thereby enhancing autophagy and eventually inducing bortezomib resistance in MM cell. Moreover, knockdown of Beclin‐1 profoundly inhibits NEK2‐mediated bortezomib resistance. These findings indicate that NEK2 induces autophagy‐mediated bortezomib resistance through stabilizing Beclin‐1 in MM cell.

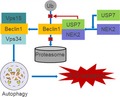

**Abbreviations**
BTZ: bortezomib
CQ: chloroquine
MM: multiple myeloma
MS: mass spectrometry
TEM: transmission electron microscopy

## 1 Introduction

Multiple myeloma (MM) is the second most frequent hematological malignancy characterized by transformed clonal plasma cells in the bone marrow (BM) microenvironment, monoclonal immunoglobulin in the blood or urine, and associated organ dysfunctions (Palumbo and Anderson, 2011). The combination of autologous stem cell transplantation (ASCT) and multiple chemotherapeutic drugs such as proteasome inhibitor [bortezomib (BTZ), carfilzomib, ixazomib], immunomodulatory drugs (thalidomide, lenalidomide, pomalidomide), alkylating agent (melphalan), and corticosteroid (dexamethasone) have significantly extended patient overall survival in MM (Palumbo and Anderson, 2011). However, MM is still a difficult‐to‐cure disease. A main reason for the treatment failure and disease relapse is the appearance of drug‐resistant subclones during therapy. A better understanding of the mechanisms by which drug resistance is caused is urgently required.

Never in mitosis‐related kinase 2 (NEK2) is a serine/threonine kinase that promotes centrosome splitting and ensures correct chromosome segregation during the G2/M phase of the cell cycle (Xia *et al.*, 2015). Previous data from our group and others have indicated that NEK2 is overexpressed in various cancers, including MM (Zhou *et al.*, 2013), cholangiocarcinoma (Kokuryo *et al.*, 2007), testicular seminomas (Barbagallo *et al.*, 2009), human breast cancer (Tsunoda *et al.*, 2009; Wang *et al.*, 2012), cervical cancer (Wang *et al.*, 2012), prostate cancer (Wang *et al.*, 2012), and colorectal cancer (Neal *et al.*, 2014; Suzuki *et al.*, 2010). Our previous studies have identified that NEK2 was the gene most strongly associated with inferior survival in MM and other cancers, suggesting that NEK2 is a potential therapeutic target in MM (Zhou *et al.*, 2013). NEK2 was found to promote cancer cell proliferation and drug resistance in MM. Subsequent mechanistic studies showed that NEK2 induces drug resistance through up‐regulation of efflux drug pumps in MM cells. NEK2 was also found to bind to USP7, a deubiquitinase that contributes to malignancy and BTZ resistance, and was stabilized by USP7 (Franqui‐Machin *et al.*, 2018). Destabilization of NEK2 by USP7 inhibitor was able to overcome resistance to BTZ in MM cells.

Autophagy is an evolutionarily conserved cellular process. During autophagy, unwanted cellular components, including proteins, lipids, entire organelles, and invading pathogens, are delivered by double‐membrane vesicles called autophagosomes to the lysosome, where they are degraded (Galluzzi and Green, 2019; Mizushima and Komatsu, 2011). This degradative process is critical for cellular homeostasis, and defects in autophagy lead to various diseases. In cancer progression, autophagy plays dual roles, either as a tumor suppressor or as a tumor progression promoter (Fu *et al.*, 2017; Guo *et al.*, 2013; Kocaturk *et al.*, 2019). Therefore, it is difficult to develop efficient treatment strategies targeting autophagy in cancer. Recent studies indicated that inhibition of autophagy enhances MM cell death induced by proteasome inhibitors (Hoang *et al.*, 2009). Intriguingly, a phase I clinical trial demonstrated that the combination of autophagy inhibitor, hydroxychloroquine, and BTZ is a useful therapeutic approach for patients with relapsed/refractory myeloma (Vogl *et al.*, 2014). Subsequently, several groups further determined that inhibition of autophagy by other mechanisms significantly increases cell death in MM cell in response to BTZ or carfilzomib, which is a second generation of approved proteasome inhibitor (Jarauta *et al.*, 2016; Lu *et al.*, 2018; Roy *et al.*, 2016; Zhang *et al.*, 2015, 2014, 2018). In view of former studies, we hypothesized that inhibition of autophagy overcomes NEK2‐mediated BTZ resistance.

In this study, we explored whether NEK2 promotes autophagy in MM cells and tested whether inhibition of autophagy overcomes NEK2‐induced BTZ resistance. Moreover, we also uncovered the underlying mechanism by which NEK2 enhances autophagy in MM cells.

## 2 Materials and methods

### 2.1 Clinical samples

Bone marrow specimens derived from healthy donors (HD; *n* = 6), and MM patients who were newly diagnosed (*n* = 9) and relapsed (*n* = 7) were obtained from Xiangya Hospital, the Second Xiangya Hospital, the Third Xiangya Hospital of Central South University during the period of 2017 to 2019. Firstly, monocytes were isolated from freshly received BM specimen (1 mL) by lymphocyte separation medium (TBD, Tianjin, China), and then, CD138^+^ cells were isolated by using CD138 antibody‐conjugated magnetic beads (Miltenyi Biotec, Bergisch Gladbach, Germany). All CD138^+^ cells were fixed in methanol for immunofluorescence until use. The Ethics Committee of Cancer Research Institute at the Central South University approved this study according to the Declaration of Helsinki. Informed written consent was obtained from all individual participants included in the study. The information of specimens is described in Table S1.

### 2.2 Reagents and antibodies

General laboratory reagents were obtained from Thermo Fisher Scientific (Waltham, MA, USA) and Sigma‐Aldrich (Saint Louis, MO, USA). Antibodies against LC3B (#2775S), PARP (#9542S), cleaved caspase‐3 (#9661S), USP7 (#4833S), ubiquitin (#3933S), HA‐tag (#3724S), Vps15 (#14580S), and Vps34 (#3811S) were obtained from Cell Signaling Technology (Danvers, MA, USA). Antibodies against NEK2 (sc‐55601), Beclin‐1 (sc‐48381), and phosphorylated mTOR (Ser 2448) (sc‐293133) were obtained from Santa Cruz Biotechnology, Inc (St Louis, MO, USA). β‐Actin antibody (66009‐1‐1g) and Flag antibody (20543‐1‐AP) were purchased from Proteintech Group (Rosemont, IL, USA). Phosphorylated Beclin‐1 (S90/93/96) antibody (#13232) and HRP‐conjugated secondary goat anti‐rabbit (#L3012) and goat anti‐mouse (#L3032) antibodies were purchased from Signalway Antibody (College Park, MD, USA). Alexa Fluor 488‐conjugated donkey anti‐rabbit second antibody (#A21202), Alexa Fluor 594‐conjugated donkey anti‐mouse second antibody (#A21207), and UltraPure™ LMP agarose were purchased from Thermo Fisher Scientific. IPKine HRP goat anti‐mouse IgG LCS (#A250112) was obtained from Abbkine Scientific Co (Wuhan, China).

### 2.3 Cell culture

Human MM cell lines, including KMS11, RPMI 8226, ARP1, KMS28‐PE, U266, MM1.S, and XG1, were cultured at 37 °C and 5% CO_2_ in RPMI 1640 medium (Gibco, Thermo Fisher Scientific) supplemented with 10% heat‐inactivated FBS (Gibco, Thermo Fisher Scientific) and 1% penicillin and streptomycin (Gibco, Thermo Fisher Scientific).

### 2.4 Plasmids and virus production

Human *NEK2* cDNA sequence was amplified and then cloned into the pCDH‐CMV‐MCS‐EF1‐copRFP lentiviral vector. Short hairpin RNA sequences targeting human *NEK2* or *BECN1* were obtained from the RNAi consortium collection (MISSION® shRNA; Sigma, http://www.sigmaaldrich.com). shRNAs were annealed and ligated into pLKO‐tet‐on lentiviral vector. Recombinant lentivirus was produced by transient transfection of 293T cells. After lentivirus transduction, NEK2‐overexpressing (NEK2‐OE) MM cells were purified by flow cytometry sorting, and MM cells expressing NEK2‐shRNA RNA or BECN1‐shRNA were selected with puromycin (1 μg·L^−1^). All primer sequences are listed in Table S2.

### 2.5 Western blotting

Western blot analysis was performed as described previously (Gu *et al.*, 2015). Briefly, total proteins were extracted using RAPI strong buffer (Auragene Bioscience, Changsha, China) with freshly added proteinase inhibitor. Proteins were then separated by 10–12% SDS/PAGE and transferred to polyvinylidene fluoride (Millipore‐Sigma). The membranes were blocked with 5% nonfat dry milk in Tris‐buffered saline (TBS) containing 0.05% Tween‐20 (TBST) prior to incubation with primary antibodies overnight at 4 °C. Respective HRP‐conjugated secondary antibodies were added, and protein signals were developed with SuperSignal™ West Femto Maximum Sensitivity Substrate (Thermo Fisher Scientific). The developed images were obtained and analyzed using ChemiDox XRS Chemiluminescence imaging system (Bio‐Rad, Hercules, CA, USA).

### 2.6 Immunofluorescence analysis

4 × 10^4^ CD138^+^ cells were spun down on glass slides and then fixed with methanol for 15 min at −20 °C. NEK2 antibodies (1 : 100 final dilution) and LC3B antibodies (1 : 200 final dilution) were diluted in antibody dilution buffer (TBS, 0.1% Triton X‐100, 1% BSA). The diluted antibodies were dripped on glass slides and incubated overnight at 4 °C. Then, slides were washed with TBST for three times followed by incubation of secondary antibodies conjugated with Alexa Fluor 488 donkey anti‐rabbit or Alexa Fluor 594 donkey anti‐mouse for 1 h at room temperature (protect slides from light). The nuclei were labeled with DAPI (Solarbio, Beijing, China). Fluorescence was observed under fluorescence microscope.

### 2.7 Co‐immunoprecipitation

Co‐immunoprecipitation (Co‐IP) was performed based on previous publication (Gu *et al.*, 2017). Briefly, total proteins were extracted with IP lysis buffer (Thermo Fisher Scientific). NEK2 antibodies (1 : 50 final dilution) or Beclin‐1 antibodies (1 : 50 final dilution) were added and incubated with cell lysate overnight at 4 °C. The same cell lysate incubated with normal mouse IgG was set as control, then followed by protein A Dynabeads (Thermo Fisher Scientific) incubation for 2 h at 4 °C. The beads were washed three times with PBST. The pulled‐down proteins were obtained and examined by western blotting as described above.

### 2.8 Soft agar colony formation assay

Soft agar colony formation assay was performed based on previous report (Yang *et al.*, 2014). Cells were planted in 12‐well plate (2000 cells/well) and fed with RPMI 1640 complete medium in the presence or absence of drugs twice every week. One colony was defined if more than 40 cells were observed. Plates were imaged, and colonies were enumerated using imagej software (NIH, Bethesda, MD, USA). Each sample was repeated three times.

### 2.9 Cell viability

Cell counts and viable cell number were determined using trypan blue staining as previously described (Xia *et al.*, 2017). Cell viability was calculated by dividing viable cells by total cell number, each sample was done in triplicate.

### 2.10 Flow cytometry

Apoptotic cells were labeled by FITC‐conjugated Annexin V (US Everbright Inc, San Francisco, CA, USA). Dead cells were labeled by PI (US Everbright Inc). Cell staining was performed according to the manufacturer’s protocol. Cells were then analyzed on LSR II cytometer (BD Biosciences, San Jose, CA, USA), and the results were analyzed by using flowjo software (BD Biosciences).

### 2.11 Autophagy detection with DALGreen staining

DALGreen staining was performed based on previous publication with some modifications (Iwashita *et al.*, 2018). A total of 200 000 cells were planted in 24‐well plate and cultured at 37 °C with 1 mL of 0.5 μm DALGreen (Dojindo Laboratories, Minato‐ku, Tokyo, Japan) working solution for 30 min. After the cells were washed twice with RPMI 1640 medium, they were observed under fluorescence microscope.

### 2.12 Tumor xenografts in mice

Mouse experiment was performed under the protocol approved by the Institutional Animal Care and Local Veterinary Office and Ethics Committee of the Central South University, China (animal experimental license number: 2019SYDW0115). NEK2‐OE KMS11 MM cell line transduced with BECN1‐shRNA or scramble‐shRNA (1 × 10^6^ cells in 200 μL RPMI 1640 medium) was injected subcutaneously into the left abdomen of immunocompromised B‐NDG (NOD‐Prkdc^scid^ IL2rg^tm1^/Bcgen) mouse (Biocytogen, Beijing, China), which lacks T cell, B cell, and NK cell. Tumor burden was monitored by tumor volume. shRNA expression was induced by the addition of doxycycline into the drinking water following the previous publication (Zhou *et al.*, 2013). The mice were euthanized when a humane endpoint was reached.

### 2.13 Statistical analysis

All data were shown as means ± standard error. Student’s *t*‐test was used to compare two experimental groups. A value of *P* < 0.05 was considered to be significant.

## 3 Results

### 3.1 Inhibition of autophagy sensitizes high NEK2 myeloma cells to bortezomib

**Figure 1:**
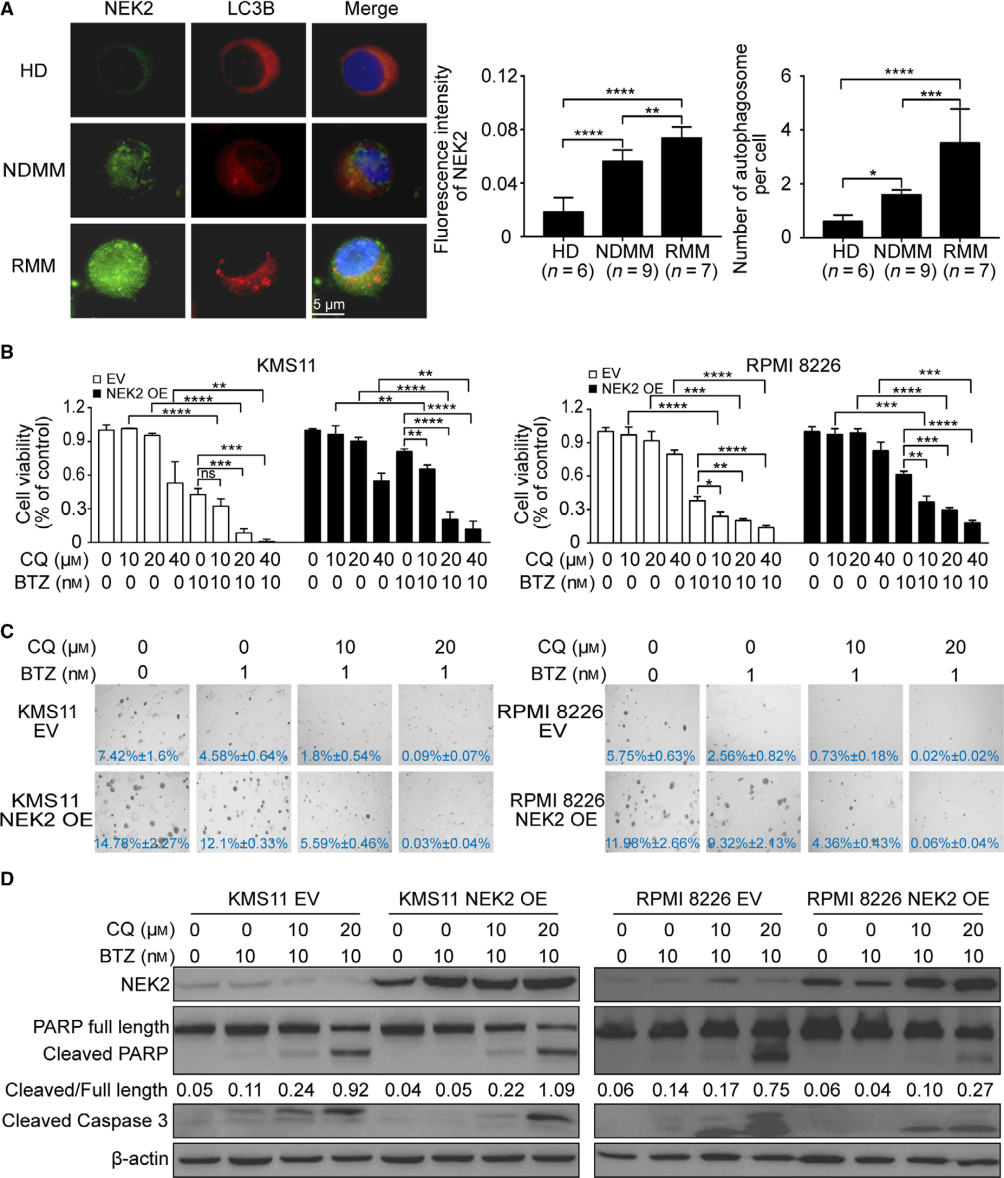
Inhibition of autophagy sensitizes NEK2‐OE MM cells to BTZ. (A) Representative images of immunofluorescence analysis for NEK2 (green) and LC3B (red) protein expression in CD138^+^ cells derived from HD (*n* = 6), NDMM patients (*n* = 9), and RMM patients (*n* = 7). (B) KMS11 EV, KMS11 NEK2‐OE, RPMI 8226 EV, and RPMI 8226 NEK2‐OE MM cells were treated with different doses of CQ (0, 10 μm, 20 μm, 40 μm) in combination or not with BTZ (10 nm), and cell viability was examined 48 h later. (C) Clonogenic analysis of KMS11 EV, KMS11 NEK2‐OE, RPMI 8226 EV, and RPMI 8226 NEK2‐OE MM cells after treatment with CQ (0, 10 μm, 20 μm) in combination or not with BTZ (1 nm), respectively (4×). (D) Western blots of full‐length PARP, cleaved PARP, cleaved caspase‐3, NEK2, and β‐actin in KMS11 EV, KMS11 NEK2‐OE, RPMI 8226 EV, and RPMI 8226 NEK2‐OE MM cells after treatment with CQ (0, 10 μm, 20 μm) in combination with or not with BTZ (10 nm). The ratio of integrated density between cleaved PARP and full‐length PARP was shown under the band of PARP. Scale bar: 5 μm. ^ns^ *P *> 0.05, *P < 0.05, **P < 0.01, ***P < 0.001, ****P < 0.0001. Significance was determined by Student’s *t*‐test. Error bars indicate SD.

Several groups have reported that increased autophagy contributes to BTZ resistance, and targeting autophagy is a useful strategy to overcome BTZ resistance in MM (Vogl *et al.*, 2014; Zhang *et al.*, 2015, 2014, 2018). Our previous studies demonstrated high NEK2 is associated with poor prognosis in MM, and overexpression of NEK2 promotes BTZ resistance (Zhou *et al.*, 2013). We thus speculated that autophagy might be involved in NEK2‐mediated BTZ resistance. To explore the correlation between NEK2 and autophagy during MM progression, immunofluorescence was performed to detect the expression of NEK2 and LC3B in CD138^+^ cells derived from HD (*n* = 6), newly diagnosed MM (NDMM) patients (*n* = 9), and relapsed MM (RMM) patients (*n* = 7). It has been widely accepted that the presence of LC3B in autophagosomes and the conversion of LC3B‐Ⅰ to the lower migrating form, LC3B‐Ⅱ, are indicators of autophagy (Kabeya *et al.*, 2004). As shown in Fig. 1A, NEK2 is profoundly elevated along with the disease progression in MM; simultaneously, the number of autophagosomes labeled by LC3B is also increased.

We subsequently asked whether inhibition of autophagy overcomes NEK2‐mediated BTZ resistance in MM cells. To answer this question, we overexpressed NEK2 by lentivirus‐mediated NEK2‐cDNA transfection in both KMS11 and RPMI 8226 MM cell lines. Those cells transduced with empty vector were used as controls. KMS11 or RPMI 8226 MM cells overexpressing NEK2 were treated with BTZ in combination or not with different doses of a specific inhibitor of autophagy, chloroquine (CQ), for 2 days. Consistent with our previous report (Zhou *et al.*, 2013), overexpression of NEK2 promoted BTZ resistance in both KMS11 and RPMI 8226; however, a combination of BTZ and CQ profoundly increased cell death compared with the drug BTZ or CQ alone in NEK2‐OE MM cell lines (Fig. 1B). The clonogenic soft agar assay was performed to confirm the synergistic effect between BTZ and CQ. The colonies were dramatically decreased in NEK2‐OE KMS11 or RPMI 8226 MM cell line treated with the combination of BTZ plus CQ, but showed only a slight decrease in those MM cell lines treated with BTZ or CQ alone (Fig. 1C). The synergistic effect between BTZ and CQ was further evidenced by increased cleavage of caspase‐3 and PARP in NEK2‐OE KMS11 or RPMI 8226 MM cell line in response to combination of BTZ and CQ compared with those cells treated with BTZ alone (Fig. 1D).

### 3.2 NEK2 enhances autophagy in myeloma cells

**Figure 2:**
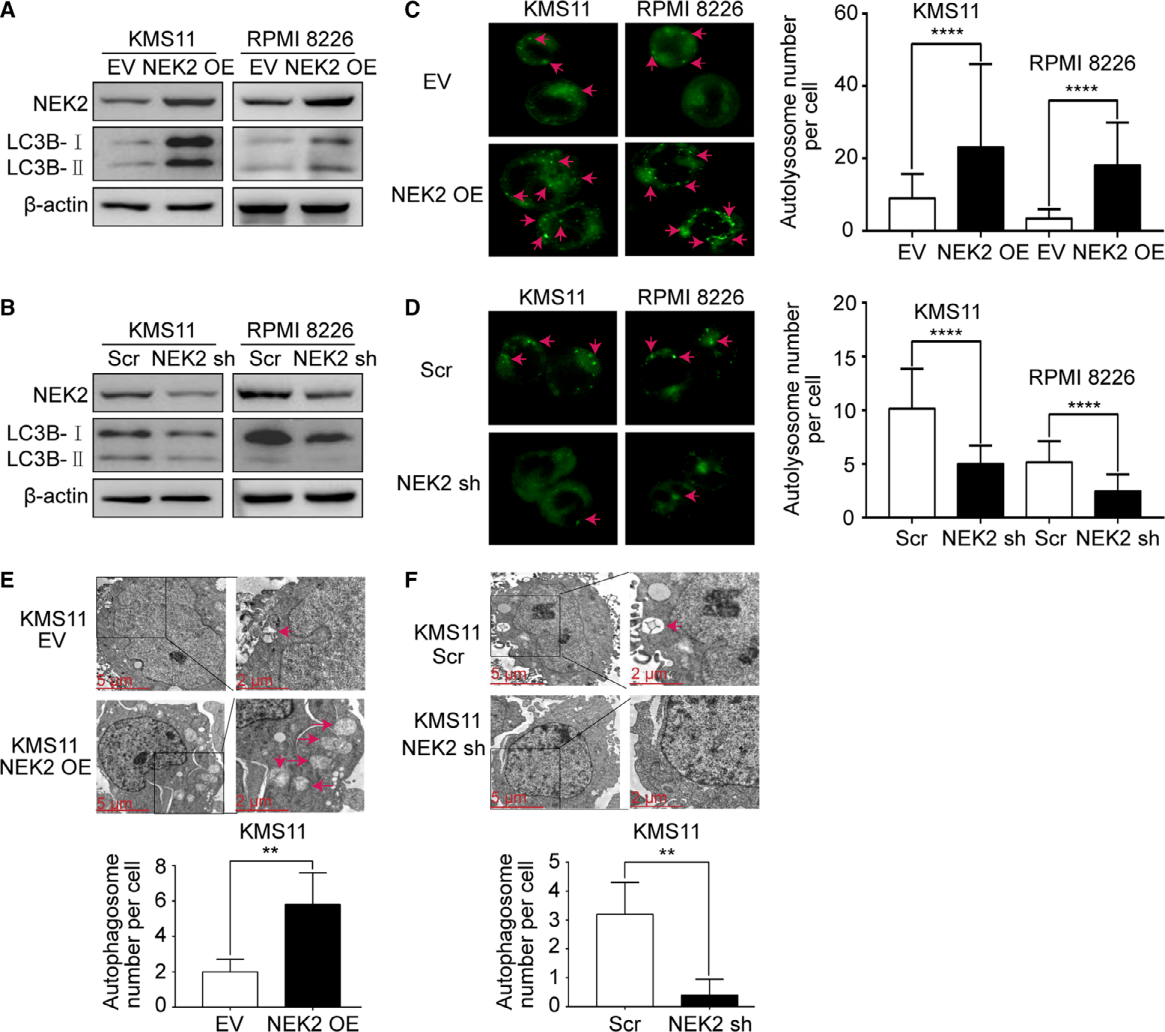
NEK2 modulates autophagy in MM cells. (A) Western blots of NEK2, LC3B, and β‐actin in KMS11 EV, KMS11 NEK2‐OE, RPMI 8226 EV, and RPMI 8226 NEK2‐OE MM cells. (B) Western blots of NEK2, LC3B, and β‐actin in KMS11 Scr, KMS11 NEK2‐shRNA, RPMI 8226 Scr, and RPMI 8226 NEK2‐shRNA MM cells. (C) KMS11 EV, KMS11 NEK2‐OE, RPMI 8226 EV, and RPMI 8226 NEK2‐OE MM cells imaging with 0.5 μm DALGreen staining. (D) KMS11 Scr, KMS11 NEK2‐shRNA, RPMI 8226 Scr, and RPMI 8226 NEK2‐shRNA imaging with 0.5 μm DALGreen staining. (E) TEM analysis of autophagosomes in KMS11 EV and KMS11 NEK2‐OE MM cells. (F) TEM analysis of autophagosomes in KMS11 Sr and KMS11 NEK2‐shRNA MM cells. Scale bar: 5 or 2 μm. **P < 0.01, ****P < 0.0001. Significance was determined by Student’s *t*‐test. Error bars indicate SD.

The data presented in Fig. 1 suggest that elevation of NEK2 is associated with autophagy activity in MM drug resistance. The conversion of LC3B‐Ⅰ to LC3B‐Ⅱ has been widely accepted as a marker for autophagy. As shown in Fig. 2A,B, overexpression of NEK2 increases LC3B‐Ⅱ in both KMS11 and RPMI 8226 MM cell lines by western blotting, while knockdown of NEK2 by a doxycycline‐inducible lentiviral expression system containing NEK2‐shRNA reduces LC3B‐Ⅱ in the same MM cell lines. To examine autophagy level, DALGreen, a small green hydrophobic molecule utilized for the imaging of autolysosomes, was introduced into MM cells. The DALGreen‐positive cells were significantly increased in NEK2‐OE KMS11 and RPMI 8226 MM cell lines but reduced in NEK2‐silenced KMS11 and RPMI 8226 MM cells (Fig. 2C,D). Transmission electron microscopy (TEM) was further performed to detect autophagosomes in NEK2‐overexpressed or knocked‐down KMS11 MM cells. Consistently, autophagosomes were increased in NEK2‐OE KMS11 and decreased in NEK2‐silenced KMS11 (Fig. 2E,F).

### 3.3 NEK2 activates autophagy through up‐regulating Beclin‐1 at protein level

**Figure 3:**
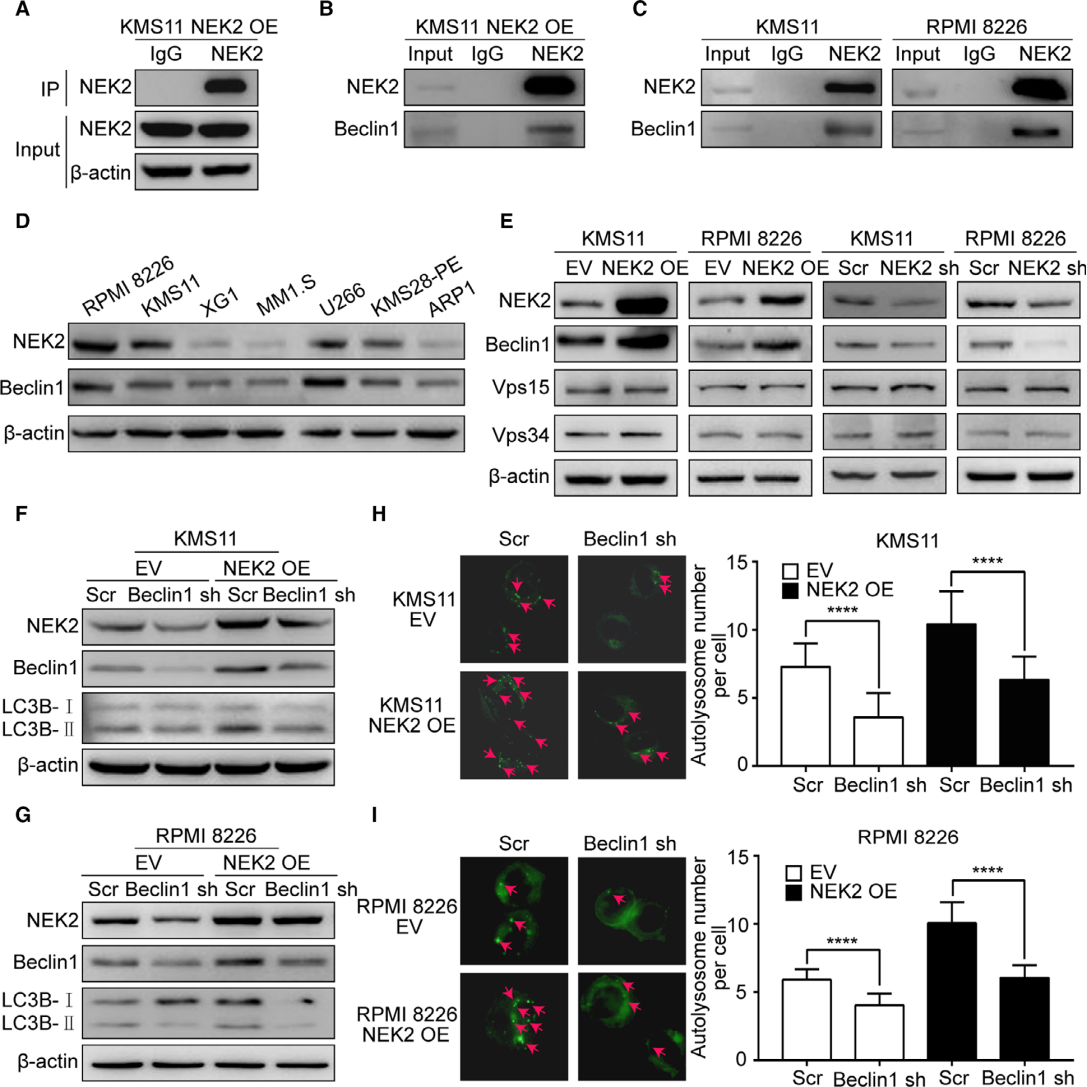
NEK2 enhances autophagy through elevating Beclin‐1 at protein level. (A) NEK2 antibody was used to pull down NEK2 in KMS11 NEK2‐OE MM cells, and the efficiency of immunoprecipitation was confirmed by western blotting before MS. The lysates before IP were used as a positive control, and normal mouse IgG pulled‐down proteins, as a negative control. (B) NEK2 antibody was used to pull down NEK2 in KMS11 NEK2‐OE MM cells, and its interacting proteins were analyzed by western blotting. (C) NEK2 antibody was used to pull down NEK2 in KMS11 (left) and RPMI 8226 (right) MM cells, and its interacting proteins were analyzed by western blotting. (D) Western blots of NEK2, Beclin‐1, and β‐actin in RPMI 8226, KMS11, XG1, MM1.S, U266, KMS28‐PE, ARP1 MM cells. (E) Western blots of NEK2, Beclin‐1, Vps15, Vps34, and β‐actin in KMS11 EV, KMS11 NEK2‐OE, RPMI 8226 EV, RPMI 8226 NEK2‐OE, KMS11 Scr, KMS11 NEK2‐shRNA, RPMI 8226 Scr, RPMI 8226 NEK2‐shRNA MM cells. (F) Western blots of NEK2, Beclin‐1, LC3B, and β‐actin in KMS11 EV + Scr, KMS11 EV + BECN1‐shRNA, KMS11 NEK2‐OE + Scr, KMS11 NEK2‐OE + BECN1‐shRNA MM cells. (G) Western blots of NEK2, Beclin‐1, LC3B, and β‐actin in RPMI 8226 EV + Scr, RPMI 8226 EV + BECN1‐shRNA, RPMI 8226 NEK2‐OE + Scr, RPMI 8226 NEK2‐OE + BECN1‐shRNA MM cells. (H) KMS11 EV + Scr, KMS11 EV + BECN1‐shRNA, KMS11 NEK2‐OE + Scr, KMS11 NEK2‐OE + BECN1‐shRNA MM cells imaging with 0.5 μm DALGreen staining. (I) RPMI 8226 EV + Scr, RPMI 8226 EV + BECN1‐shRNA, RPMI 8226 NEK2‐OE + Scr, RPMI 8226 NEK2‐OE + BECN1‐shRNA MM cells imaging with 0.5 μm DALGreen staining. ****P < 0.0001. Significance was determined by Student’s *t*‐test. Error bars indicate SD.

To determine the underlying mechanisms by which NEK2 promotes autophagy in MM cells, mass spectrometry (MS) was performed to identify NEK2‐interacting proteins in NEK2‐OE KMS11 MM cell line using NEK2 antibodies (Fig. S1A). Proteins pulled down by normal mouse IgG were identified as nonspecific binding. Western blots confirmed NEK2 was effectively pulled down by NEK2 antibodies (Fig. 3A). We obtained 156 proteins bound to NEK2, which were not observed in proteins pulled down by normal mouse IgG (data not shown). Interestingly, Beclin‐1, which interacts with Vps15‐Vps34 complex to play a crucial role in the initiation of autophagy, was found to bind to NEK2. The interaction between NEK2 and Beclin‐1 was confirmed by Co‐IP in NEK2‐OE KMS11 MM cell line (Fig. 3B). To further confirm the interaction between NEK2 and Beclin‐1, Co‐IPs of endogenous NEK2 in KMS11 and RPMI 8226 MM cell lines were performed using NEK2 antibodies. Western blots were applied to detect NEK2 and Beclin‐1. As shown in Fig. 3C, both NEK2 and Beclin‐1 are detected in NEK2 antibody‐immunoprecipitated proteins but not in IgG control. As the sizes of NEK2 protein band and Beclin‐1 protein band in SDS/PAGE are similar to IgG heavy chain, we used HRP‐conjugated second antibody only interacting with IgG light chain but not with heavy chain in Co‐IP experiments. In addition, we found an interesting phenomenon that the protein level of NEK2 was positively related to that of Beclin‐1 in seven MM cell lines (Figs 3D and S1B). We thus examined whether Beclin‐1 expression is regulated by NEK2. Western blots showed Beclin‐1 was significantly increased in NEK2‐OE KMS11 and RPMI 8226 MM cell lines compared with the controls, while silence of NEK2 decreased Beclin‐1 (Fig. 3E). We did not observe significant change in Beclin‐1 at mRNA level in the same MM cell lines (Fig. S1C). In addition, the expression of Beclin‐1‐interacting proteins Vps15 and Vps34 was not affected by NEK2 (Fig. 3E). Several studies have documented that autophagy is enhanced by phosphorylation of Beclin‐1 at Ser90, Ser93, and Ser96 (Menon and Dhamija, 2018). NEK2 is a serine/threonine kinase that was found to phosphorylate and then activate several oncogenes. The changes in Beclin‐1 phosphorylation were not observed in NEK2‐OE KMS11 MM cell line (Fig. S1D). Furthermore, the phosphorylation of mTOR, the upstream and negative regulator of Beclin‐1‐Vps15‐Vps34 complex (Navé *et al.*, 1999), was not affected by NEK2 at ser‐2448, which is the active site of mTOR (Fig. S1D).

To determine whether Beclin‐1 mediates NEK2‐enhanced autophagy, we knocked down Beclin‐1 in NEK2‐OE MM cell lines by a doxycycline‐inducible lentiviral expression system containing BECN1‐shRNA. These cell lines were exposed to doxycycline for 2 days, and then, western blots were performed to detect the expression of NEK2, Beclin‐1, and LC3B‐Ⅰ/Ⅱ. Beclin‐1 protein was clearly decreased in MM cell lines expressing BECN1‐shRNA (Fig. 3F,G). As expected, LC3B‐Ⅱ was also decreased in NEK2‐OE KMS11 and RPMI 8226 MM cell lines following the silence of Beclin‐1, indicating that NEK2 enhanced autophagy was attenuated by knockdown of Beclin‐1 (Fig. 3F,G). In addition, the number of autolysosomes labeled by DALGreen was significantly decreased in response to down‐regulation of Beclin‐1 in NEK2‐OE KMS11 and RPMI 8226 MM cell lines (Fig. 3H,I). Therefore, we conclude that NEK2 enhances autophagy through up‐regulating Beclin‐1 at protein level.

### 3.4 NEK2 stabilizes Beclin‐1 by USP7‐mediated deubiquitination

**Figure 4:**
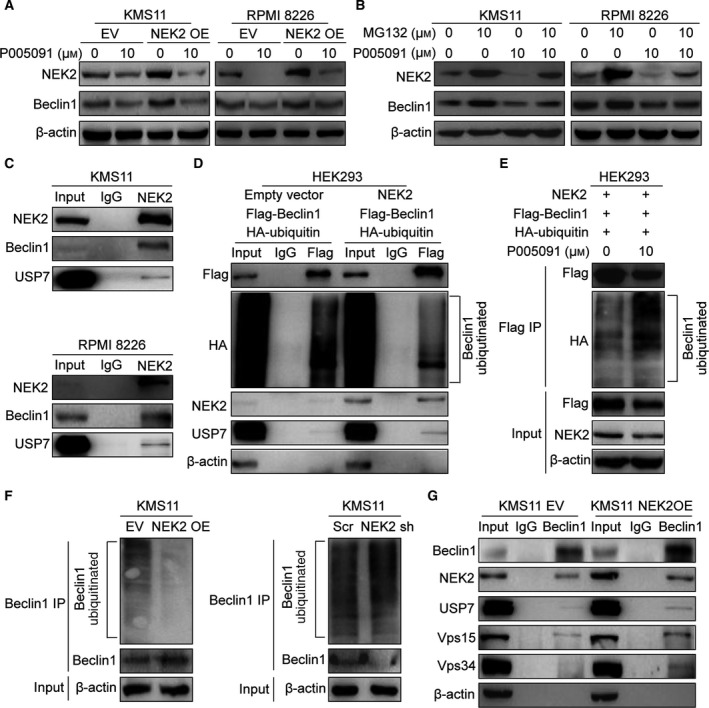
NEK2 stabilizes Beclin‐1 through USP7‐mediated deubiquitination. (A) KMS11 EV, KMS11 NEK2‐OE, RPMI 8226 EV, and RPMI 8226 NEK2‐OE MM cells were treated with 10 μm P005091 for 24 h, and the levels of NEK2 and Beclin‐1 were analyzed by western blotting. (B) KMS11 and RPMI 8226 MM cells were treated with MG132 (10 μm) alone for 1 h, or in combination with P005091 (10 μm) for an additional 9 h. Cells were lysed, and the levels of NEK2 and Beclin‐1 were analyzed by western blotting. (C) Endogenous NEK2 was pulled down by NEK2 antibodies in KMS11 (up) and RPMI 8226 (bottom) MM cells, NEK2‐interacting proteins were analyzed by western blotting. The lysates before IP were used as a positive control, and normal mouse IgG pulled‐down proteins, as a negative control. (D) HEK293 cells were transfected with HA‐ubiquitin vector, Beclin‐1‐Flag vector, and NEK2 overexpression vector or empty vector. After 48 h, cells were lysed, Beclin‐1‐flag fusion protein and its interacting proteins were pulled down by flag antibody‐conjugated beads, and the ubiquitination level of Beclin‐1 and its interacting proteins were analyzed by western blotting. (E) HEK293 cells were transfected with Beclin‐1‐Flag vector, HA‐ubiquitin vector, NEK2 overexpression vector; after 48 h, cells were treated with USP7 inhibitor P005091 (10 μm) for 10 h; cells were lysed; Beclin‐1‐flag fusion protein and its interacting proteins were pulled down by flag antibody‐conjugated beads; and the ubiquitination level of Beclin‐1 was analyzed by western blotting. (F) Endogenous Beclin‐1 was pulled down by Beclin‐1 antibodies in KMS11 EV, KMS11 NEK2‐OE, KMS11 Scr, and KMS11 NEK2‐shRNA MM cells, and Beclin‐1, ubiquitinated Beclin‐1, and β‐actin were analyzed by western blotting. (G) Beclin‐1 antibody was used to pull down Beclin‐1 in KMS11 EV and KMS11 NEK2‐OE MM cells, and western blots were performed to analyze the levels of Beclin‐1, NEK2, Vps15, Vps34, USP7, and β‐actin.

Beclin‐1 can be stabilized by several deubiquitinases, including USP19, USP13, and USP10 (Jin *et al.*, 2016; Liu *et al.*, 2011). In this study, we showed proteasome inhibitor, BTZ, elevated Beclin‐1 at protein level in MM cells (Fig. S2A). Addition of proteasome inhibitor MG132 to NEK2‐silenced MM cells profoundly increased Beclin‐1 protein (Fig. S2B). These data suggested that NEK2 might elevate Beclin‐1 through blocking proteasomal degradation. We have reported that USP7 binds and stabilizes NEK2 protein (Franqui‐Machin *et al.*, 2018). NEK2 interacts with both USP7 and Beclin‐1 in MM cells, and we thus hypothesized that NEK2 stabilizes Beclin‐1 via interacting with USP7. To address this hypothesis, NEK2‐OE KMS11 and RPMI 8226 MM cell lines were treated with P005091, a USP7 inhibitor that selectively binds the USP7 active site and inhibits its activity, overnight at 10 μm. As shown in Fig. 4A, treatment with P005091 decreases Beclin‐1 in both NEK2‐OE MM cells and controls, suggesting that Beclin‐1 is stabilized by USP7. Since USP7 stabilizes its targets by preventing proteasomal degradation, we tested whether Beclin‐1 depletion by P005091 was dependent on proteasome activity. KMS11 and RPMI 8226 MM cell lines were treated with MG132 (10 μm) alone for 1 h or in a combination with P005091 (10 μm) for additional 9 h. Western blots showed P005091 alone depleted Beclin‐1; however, addition of MG132 blocked this depletion, indicating that USP7 stabilizes Beclin‐1 through preventing ubiquitination–proteasomal degradation of Beclin‐1 (Fig. 4B).

We subsequently examined the interaction between NEK2, Beclin‐1, and USP7 in MM cells. Endogenous NEK2 was pulled down by NEK2 antibodies in both KMS11 and RPMI 8226 MM cell lines. Western blots showed NEK2 was bound to Beclin‐1 and USP7, suggesting that a complex containing NEK2, Beclin‐1, and USP7 was persistent in KMS11 and RPMI 8226 MM cell lines (Fig. 4C). To determine whether increased deubiquitination of Beclin‐1 is induced by NEK2, HEK293 cells were transfected with HA‐ubiquitin vector, Beclin‐1‐Flag vector, and NEK2 overexpression vector or empty vector. Beclin‐1 was immunoprecipitated with flag antibody‐conjugated beads. Less ubiquitinated Beclin‐1 was observed in NEK2‐OE HEK293 cells compared with those cells transfected with empty vector. Furthermore, western blots detected more USP7 bound to Beclin‐1 in NEK2‐OE HEK293 cells (Fig. 4D). HEK293 cells transfected with HA‐ubiquitin vector, Beclin‐1‐Flag vector, and NEK2 overexpression vector were treated with P005091 and then pulled down Beclin‐1 by flag antibody‐conjugated beads. Western blots showed ubiquitinated Beclin‐1 was increased in response to P005091 treatment (Fig. 4E). We also examined the ubiquitination level of endogenous Beclin‐1 in NEK2‐OE and NEK2 knocked‐down KMS11 MM cell lines by Co‐IP. Endogenous Beclin‐1 was pulled down by Beclin‐1 antibodies. Figure 4F shows overexpression of NEK2 decreases the ubiquitination level of Beclin‐1, while silence of NEK2 enhances the ubiquitination level of Beclin‐1. To explore whether NEK2 promotes the formation of Beclin‐1‐Vps15‐Vps34 complex in MM cells, we pulled down Beclin‐1 using Beclin‐1 antibodies in both NEK2‐OE KMS11 MM cells and controls. As shown in Fig. 4G, increased Vps15 and Vps34 are obtained in Beclin‐1 antibody‐immunoprecipitated proteins derived from NEK2‐OE KMS11 MM cells compared with controls, indicating that overexpression of NEK2 promotes the formation of Beclin‐1‐Vps15‐Vps34 complex. Taken together, our data demonstrated that NEK2 up‐regulates Beclin‐1 through USP7‐mediated deubiquitination, thereby promoting autophagy.

### 3.5 Knockdown of Beclin‐1 prevents NEK2‐mediated bortezomib resistance in myeloma cells

**Figure 5:**
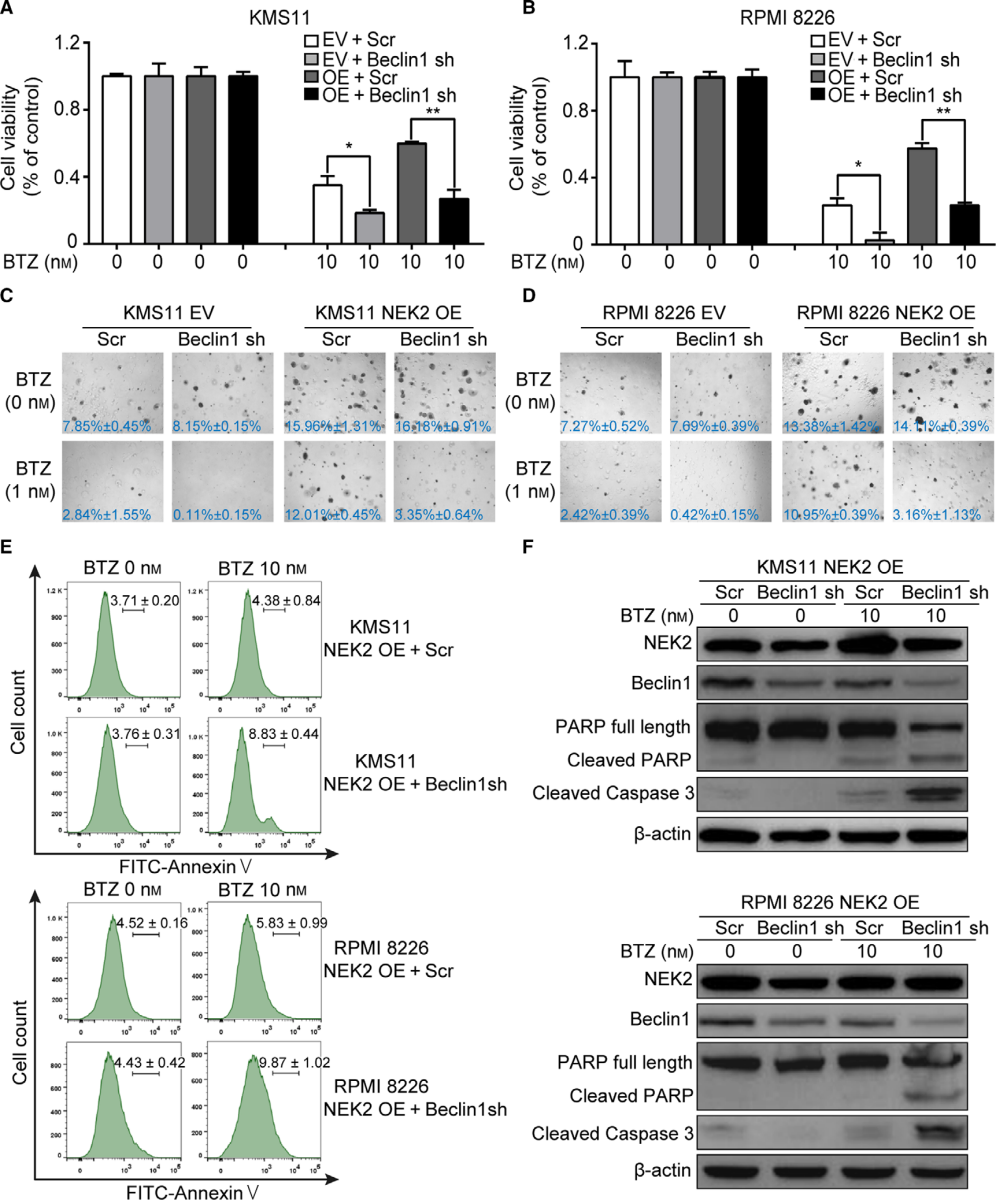
Knockdown of Beclin‐1 blocks NEK2‐mediated BTZ resistance in MM cells. (A) KMS11 EV + Scr, KMS11 EV + BECN1‐shRNA, KMS11 NEK2‐OE + Scr, KMS11 NEK2‐OE + BECN1‐shRNA MM cells were treated with BTZ (10 nm), cell viability was examined 48 h later. (B) RPMI 8226 EV + Scr, RPMI 8226 EV + BECN1‐shRNA, RPMI 8226 NEK2‐OE + Scr, RPMI 8226 NEK2‐OE + BECN1‐shRNA MM cells were treated with BTZ (10 nm), cell viability was examined 48 h later. (C) Clonogenic analysis of KMS11 EV + Scr, KMS11 EV + BECN1‐shRNA, KMS11 NEK2‐OE + Scr, KMS11 NEK2‐OE + BECN1‐shRNA MM cells after treatment with BTZ (1 nm), respectively (4×). (D) Clonogenic analysis of RPMI 8226 EV + Scr, RPMI 8226 EV + BECN1‐shRNA, RPMI 8226 NEK2‐OE + Scr, RPMI 8226 NEK2‐OE + BECN1‐shRNA MM cells after treatment with BTZ (1 nm), respectively (4×). (E) Assessment of apoptosis by using FITC‐Annexin V/PI staining in KMS11 NEK2‐OE + Scr, KMS11 NEK2‐OE + BECN1‐shRNA, RPMI 8226 NEK2‐OE + Scr, RPMI 8226 NEK2‐OE + BECN1‐shRNA MM cells after treatment with BTZ (10 nm). (F) Western blots of full‐length PARP, cleaved PARP, cleaved caspase‐3, NEK2, and β‐actin in KMS11 NEK2‐OE + Scr, KMS11 NEK2‐OE + BECN1‐shRNA, RPMI 8226 NEK2‐OE + Scr, RPMI 8226 NEK2‐OE + BECN1‐shRNA MM cells after treatment with BTZ (10 nm). *P < 0.05, **P < 0.01. Significance was determined by Student’s *t*‐test. Error bars indicate SD.

Beclin‐1 was knocked down in NEK2‐OE KMS11 MM cells and controls by doxycycline‐induced expression of BECN1‐shRNA followed by BTZ treatment for 2 days. The viability of control cells showed significant reduction after BTZ treatment, while NEK2‐OE KMS11 MM cells were highly resistant to BTZ. However, knockdown of Beclin‐1 in NEK2‐OE KMS11 MM cells significantly reduced viability after treatment with BTZ, similar to control cells treated with BTZ (Fig. 5A). A similar result was observed in the RPMI 8226 MM cell line (Fig. 5B). Subsequently, clonogenic soft agar assay was performed with above MM cell lines with or without BTZ treatment. As shown in Fig. 5C,D, compared to nontreated controls, NEK2‐OE KMS11 or RPMI 8226 MM cell lines showed only a slight decrease in their capacity to form colonies, while those cells expressing BECN1‐shRNA showed a significant decrease in colony formation when treated with the same dose of BTZ, indicating that silence of Beclin‐1 prevents BTZ resistance in NEK2‐OE MM cell lines. We next examined cell apoptosis in NEK2‐OE/BECN1‐shRNA MM cell lines when treated with BTZ. Apoptotic cells were detected by flow cytometry using FITC‐conjugated Annexin V staining. Increased apoptotic cells were observed in Beclin‐1 knocked‐down MM cells compared with those cells expressing scramble‐shRNA after BTZ treatment (Fig. 5E). Western blots also showed knockdown of Beclin‐1 resulted in increase in cleaved caspase‐3 and PARP in NEK2‐OE MM cells when treated with BTZ (Fig. 5F).

### 3.6 Knockdown of Beclin‐1 sensitizes NEK2‐OE KMS11 myeloma cells to bortezomib *in vivo*

**Figure 6:**
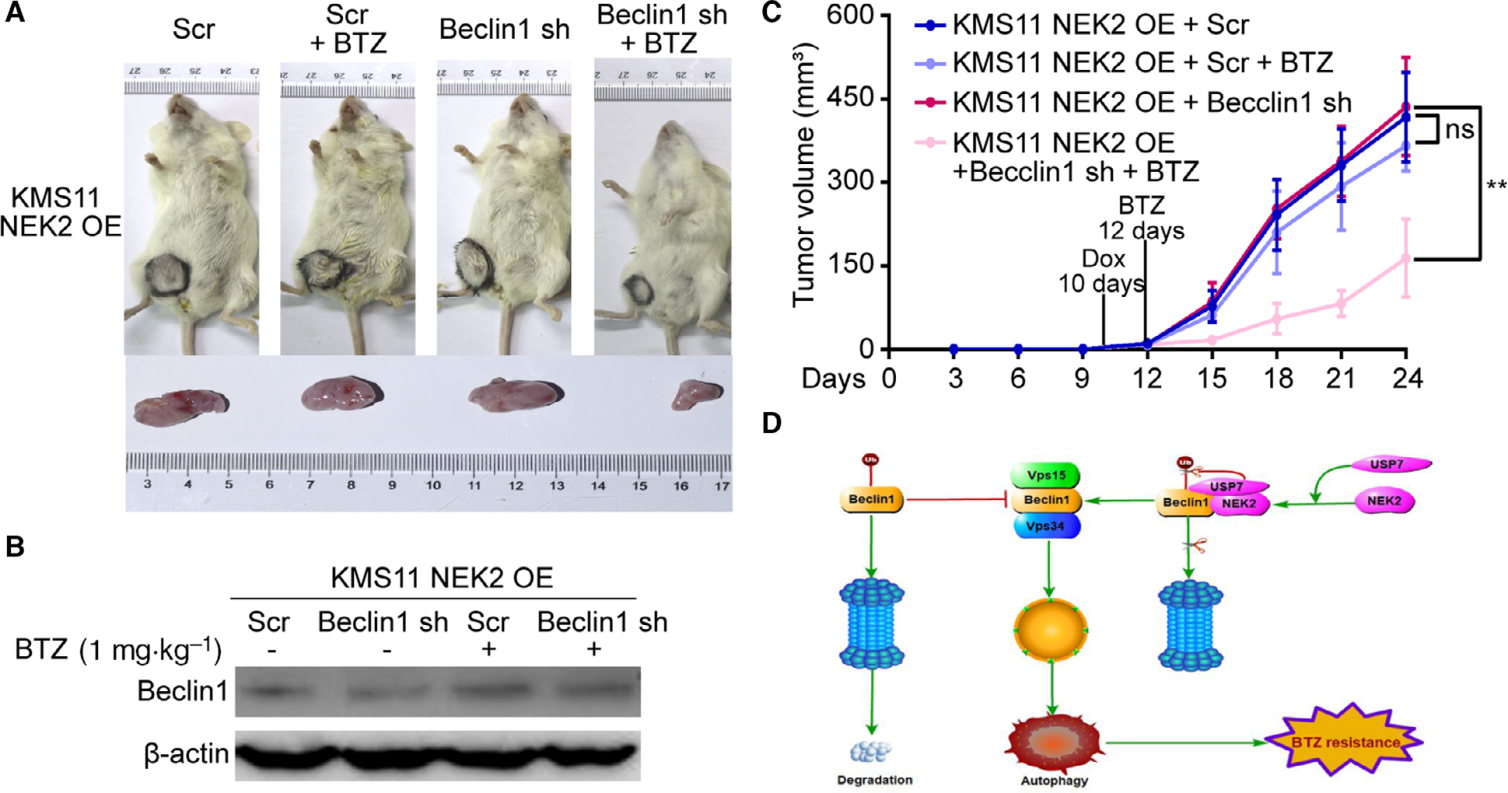
Knockdown of Beclin‐1 sensitizes NEK2‐OE KMS11 MM cells to BTZ *in vivo*. (A) KMS11 NEK2‐OE + Scr and KMS11 NEK2‐OE + BECN1‐shRNA MM cells were injected subcutaneously into the left abdomen of B‐NDG mice, and shRNA expression was induced by the addition of doxycycline to the drinking water about 10 days after injection of tumor cells. 2 days after addition of doxycycline, half mice of both groups were treated with BTZ (1 mg·kg^−1^). Differences in tumor size are shown between tumors transplanted with KMS11 NEK2‐OE + Scr or KMS11 NEK2‐OE + BECN1‐shRNA MM cells treated with or without BTZ. (B) Western blots showed Beclin‐1 is indeed knocked down in KMS11 NEK2‐OE + BECN1‐shRNA MM cells after BECN1‐shRNA expression. (C) Tumor volume assessments showed smaller tumor sizes in KMS11 NEK2‐OE + BECN1‐shRNA mice treated with BTZ than in other groups. (D) The model of our working hypothesis. ^ns^ *P*> 0.05, **P < 0.01. Significance was determined by Student’s *t*‐test. Error bars indicate SD.

NEK2‐OE KMS11 MM cells transduced with BECN1‐shRNA were injected subcutaneously into the left abdomen of 12 immunocompromised B‐NDG mice. The other 12 B‐NDG mice were injected with NEK2‐OE KMS11 MM cells transduced with scramble‐shRNA. Ten days after engraftment of the tumor cells, shRNA expression was induced by the addition of doxycycline (2 mg·mL^−1^) into the drinking water. Two days later, six mice selected randomly from the BECN1‐shRNA group or scramble‐shRNA group were treated with BTZ by intraperitoneal injection (1 mg·kg^−1^). As shown in Fig. 6A, similar tumor sizes are obtained in tumors expressing BECN1‐shRNA or scramble‐shRNA developed in nontreated mice; however, tumors expressing BECN1‐shRNA in BTZ‐treated mice are significantly smaller than those expressing scramble‐shRNA. Western blots confirmed that Beclin‐1 was indeed knocked down by addition of doxycycline in NEK2‐OE KMS11 MM cells (Fig. 6B). The volumes of tumors derived from each group were measured every 3 days after BTZ treatment. We found that knockdown of Beclin‐1 did not inhibit tumor growth, but significantly sensitized NEK2‐OE KMS11 MM cells to BTZ (Fig. 6C).

## 4 Discussion

Multiple myeloma is an incurable hematological malignancy. Relapse resulted from drug resistance is the most important cause for the death of MM patients. The mechanisms of drug resistance have been explored for several years. In this study, we confirmed NEK2 promotes BTZ resistance in MM, which is consistent with our previous data (Zhou *et al.*, 2013). Intriguingly, we also found NEK2 enhances autophagy through stabilizing Beclin‐1 by USP7‐mediated deubiquitination, which contributes to BTZ resistance in MM (Fig. 6D).

Multiple myeloma is a malignant plasma cell disease, which produces a mass of useless monoclonal immunoglobulin (Sirohi and Ray, 2004). Protein degradation systems that include ubiquitin–proteasome system and autophagy–lysosome system are essential for MM cells to maintain homeostasis. Thus, either ubiquitin–proteasome system or autophagy–lysosome system is a potential target for MM treatment. BTZ as the first proteasome inhibitor has achieved exciting treatment effect in MM therapy (Kane *et al.*, 2003). However, the development of BTZ resistance limits its long‐term utility. A previous clinical trial indicated that the combination of BTZ and autophagy inhibitor is a promising therapeutic approach for patients with RMM (Vogl *et al.*, 2014). In the present study, we found that NEK2 promotes autophagy in MM cells. Our data also showed that inhibition of autophagy by CQ sensitizes NEK2‐OE MM cell lines to BTZ. NEK2 is strongly associated with drug resistance and relapse in MM. At present, it is difficult to develop therapeutic approach through targeting NEK2 in MM because of a lack of specific NEK2 inhibitors. Thus, our study provides a potential novel treatment strategy in aggressive MM patients who express high NEK2 in tumor cells.

Autophagy is a multistep pathway requiring the interplay of numerous scaffolding and signaling molecules (Behrends *et al.*, 2010; Green and Levine, 2014).The kinase mTOR is a critical regulator of autophagy induction, with activated mTOR suppressing autophagy, and vice versa (Jung *et al.*, 2010). Beclin‐1‐Vps15‐Vps34 complex, the downstream target of mTOR, plays a central role in the initiation of autophagy (Ohashi *et al.*, 2019). The expression of Beclin‐1 is regulated at the level of transcription, translation, and post‐translational modifications (Kang *et al.*, 2011). Beclin‐1 serves not only as a key autophagic regulator with its specific interactors, but also as a potential therapeutic target in cancer (Fu *et al.*, 2013; Toton *et al.*, 2014). In this study, our MS and Co‐IP data showed NEK2 is bound to Beclin‐1. We also showed clearly that overexpression of NEK2 up‐regulates Beclin‐1 at protein level but not at mRNA expression. The phosphorylation of Beclin‐1 at Ser90, Ser93, and Ser96 was found to enhance autophagy (Menon and Dhamija, 2018). In our study, the change in phosphorylated Beclin‐1 at ser‐90, ser‐93, and ser‐96 was not observed in both NEK2‐overexpressed or down‐regulated MM cell lines. In addition, the expression of Beclin‐1‐interacting proteins Vps15 and Vps34 and the phosphorylation level of mTOR at ser‐2248, an indicator of mTOR intrinsic catalytic activity, were not regulated by NEK2 in MM cells. Importantly, knockdown of Beclin‐1 significantly inhibits NEK2‐induced autophagy in MM cells. Therefore, we conclude that NEK2 induces autophagy through up‐regulation of Beclin‐1 in MM cells.

Several deubiquitinases that include USP10, USP13, and USP19 were found to stabilize Beclin‐1 protein through deubiquitination (Boutouja *et al.*, 2017), indicating that Beclin‐1 is regulated by ubiquitin–proteasome system. In this study, the USP7 inhibitor significantly decreases Beclin‐1 in NEK2‐OE MM cells, and the decrease in Beclin‐1 mediated by USP7 inhibitor can be blocked by MG132, suggesting that USP7 is a novel Beclin‐1 regulator, which stabilizes Beclin‐1 through deubiquitination. USP7 is known to regulate drug resistance by stabilizing its targets, which include a range of oncogenes and tumor suppressors (Chauhan *et al.*, 2012; Song *et al.*, 2008), NEK2 is one of the USP7 targets. Our data indicated that NEK2 binds to both Beclin‐1 and USP7, and we thus sought to determine whether NEK2 stabilizes Beclin‐1 through USP7‐mediated deubiquitination. The Co‐IPs showed overexpression of NEK2 decreases the ubiquitination of exogenous Beclin‐1, which is dramatically increased by USP7 inhibitor in the same cells. The ubiquitination of endogenous Beclin‐1 is also modulated by NEK2 in MM cells. These findings demonstrate NEK2 stabilizes Beclin‐1 through removing ubiquitins from Beclin‐1 by USP7. Interestingly, we also found knockdown of Beclin‐1 decreases NEK2 in both NEK2‐OE MM cells and controls. Liu et al have reported that Beclin‐1 is not only regulated by USP10 and USP13, but also affects the deubiquitination activity of USP10 and USP13 (Liu *et al.*, 2011). In view of these data, we conclude that a regulatory network persists between NEK2, Beclin‐1, and USP7. Further studies are therefore necessary to uncover the underlying mechanisms by which NEK2, Beclin‐1, and USP7 regulate each other.

Previous studies have demonstrated inhibition of USP7 overcomes BTZ resistance in MM (Chauhan *et al.*, 2012). Destabilizing NEK2 by USP7 inhibitor overcomes drug resistance to proteasome inhibitor in MM. USP7 binds to NEK2 and prevents NEK2 ubiquitination resulting in NEK2 stabilization. Increased NEK2 activates the canonical NF‐κB signaling pathway through the PP1α/AKT axis, which is the downstream targets of USP7‐NEK2 axis (Franqui‐Machin *et al.*, 2018). In this study, we showed knockdown of Beclin‐1 prevents NEK2‐mediated BTZ resistance in MM both *in vitro* and *in vivo*. Thus, enhanced autophagy by up‐regulation of Beclin‐1 could be a novel mechanism by which the USP7‐NEK2 interaction induces BTZ resistance.

## 5 Conclusion

In summary, our findings demonstrate the interaction of NEK2 with USP7 enhances autophagy by stabilizing Beclin‐1 protein. Inhibition of autophagy significantly sensitizes NEK2‐OE MM cells to BTZ. Therefore, this study offers a promising novel therapeutic strategy to overcome NEK2‐induced drug resistance in MM.

## Conflict of interest

The authors declare no conflict of interest.

## Author contributions

WZ and JX designed the research. JX, YH, BM, SC, YZ, and YW performed the experiments and analyzed the data. JZ, XW, QL, CK, and JG collected clinical samples. YS, XF, YG, LQ, GL, and GA provided technical assistance. JX wrote the manuscript. WZ and FZ critically revised the manuscript. All authors read and approved the final manuscript.

## Supporting information


**Fig. S1**. NEK2 regulates Beclin‐1 at protein level but not affects its mRNA expression and phosphorylation.
**Fig. S2**. Beclin‐1 is regulated by proteasome inhibitors.
**Table S1**. Clinical characteristics of healthy donors and MM patients.
**Table S2**. The list of primer sequences.

## References

[mol212641-bib-0001] Barbagallo F , Paronetto MP , Franco R , Chieffi P , Dolci S , Fry AM , Geremia R and Sette C (2009) Increased expression and nuclear localization of the centrosomal kinase Nek2 in human testicular seminomas. J Pathol 217, 431–441. 19023884 10.1002/path.2471

[mol212641-bib-0002] Behrends C , Sowa ME , Gygi SP and Harper JW (2010) Network organization of the human autophagy system. Nature 466, 68–76. 20562859 10.1038/nature09204 PMC2901998

[mol212641-bib-0003] Boutouja F , Brinkmeier R , Mastalski T , El‐Magraoui F and Platta HW (2017) Regulation of the tumor‐suppressor BECLIN 1 by distinct ubiquitination cascades. Int J Mol Sci 18, 2541–2561. 10.3390/ijms18122541 PMC5751144 29186924

[mol212641-bib-0004] Chauhan D , Tian Z , Nicholson B , Kumar KG , Zhou B , Carrasco R , McDermott JL , Leach CA , Fulcinniti M , Kodrasov MP *et al* (2012) A small molecule inhibitor of ubiquitin‐specific protease‐7 induces apoptosis in multiple myeloma cells and overcomes bortezomib resistance. Cancer Cell 22, 345–358. 22975377 10.1016/j.ccr.2012.08.007 PMC3478134

[mol212641-bib-0005] Franqui‐Machin R , Hao M , Bai H , Gu Z , Zhan X , Habelhah H , Jethava Y , Qiu L , Frech I , Tricot G *et al* (2018) Destabilizing NEK2 overcomes resistance to proteasome inhibition in multiple myeloma. J Clin Invest 128, 2877–2893. 29863498 10.1172/JCI98765 PMC6026005

[mol212641-bib-0006] Fu LL , Cheng Y and Liu B (2013) Beclin‐1: autophagic regulator and therapeutic target in cancer. Int J Biochem Cell Biol 45, 921–924. 23420005 10.1016/j.biocel.2013.02.007

[mol212641-bib-0007] Fu YF , Liu X , Gao M , Zhang YN and Liu J (2017) Endoplasmic reticulum stress induces autophagy and apoptosis while inhibiting proliferation and drug resistance in multiple myeloma through the PI3K/Akt/mTOR signaling pathway. Oncotarget 8, 61093–61106. 28977849 10.18632/oncotarget.17862 PMC5617409

[mol212641-bib-0008] Galluzzi L and Green DR (2019) Autophagy‐independent functions of the autophagy machinery. Cell 177, 1682–1701. 31199916 10.1016/j.cell.2019.05.026 PMC7173070

[mol212641-bib-0009] Green DR and Levine B (2014) To be or not to be? How selective autophagy and cell death govern cell fate. Cell 157, 65–75. 24679527 10.1016/j.cell.2014.02.049 PMC4020175

[mol212641-bib-0010] Gu Z , Wang H , Xia J , Yang Y , Jin Z , Xu H , Shi J , De‐Domenico I , Tricot G and Zhan F (2015) Decreased ferroportin promotes myeloma cell growth and osteoclast differentiation. Cancer Res 75, 2211–2221. 25855377 10.1158/0008-5472.CAN-14-3804 PMC4946247

[mol212641-bib-0011] Gu Z , Xia J , Xu H , Frech I , Tricot G and Zhan F (2017) NEK2 promotes aerobic glycolysis in multiple myeloma through regulating splicing of pyruvate Kinase. J Hematol Oncol 10, 17–27. 28086949 10.1186/s13045-017-0392-4 PMC5237262

[mol212641-bib-0012] Guo JY , Xia B and White E (2013) Autophagy‐mediated tumor promotion. Cell 155, 1216–1219. 24315093 10.1016/j.cell.2013.11.019 PMC3987898

[mol212641-bib-0013] Hoang B , Benavides A , Shi Y , Frost P and Lichtenstein A (2009) Effect of autophagy on multiple myeloma cell viability. Mol Cancer Ther 8, 1974–1984. 19509276 10.1158/1535-7163.MCT-08-1177

[mol212641-bib-0014] Iwashita H , Sakurai HT , Nagahora N , Ishiyama M , Shioji K , Sasamoto K , Okuma K , Shimizu S and Ueno Y (2018) Small fluorescent molecules for monitoring autophagic flux. FEBS Lett 592, 559–566. 29355929 10.1002/1873-3468.12979 PMC5947577

[mol212641-bib-0015] Jarauta V , Jaime P , Gonzalo O , De Miguel D , Ramírez‐Labrada A , Martínez‐Lostao L , Anel A , Pardo J , Marzo I and Naval J (2016) Inhibition of autophagy with chloroquine potentiates carfilzomib‐induced apoptosis in myeloma cells *in vitro* and *in vivo* . Cancer Lett 382, 1–10. 27565383 10.1016/j.canlet.2016.08.019

[mol212641-bib-0016] Jin S , Tian S , Chen Y , Zhang C , Xie W , Xia X , Cui J and Wang RF (2016) USP19 modulates autophagy and antiviral immune responses by deubiquitinating Beclin‐1. EMBO J 35, 866–870. 26988033 10.15252/embj.201593596 PMC4972138

[mol212641-bib-0017] Jung CH , Ro SH , Cao J , Otto NM and Kim DH (2010) mTOR regulation of autophagy. FEBS Lett 584, 1287–1295. 20083114 10.1016/j.febslet.2010.01.017 PMC2846630

[mol212641-bib-0018] Kabeya Y , Mizushima N , Yamamoto A , Oshitani‐Okamoto S , Ohsumi Y and Yoshimori T (2004) LC3, GABARAP and GATE16 localize to autophagosomal membrane depending on form‐II formation. J Cell Sci 117, 2805–2812. 15169837 10.1242/jcs.01131

[mol212641-bib-0019] Kane RC , Bross PF , Farrell AT and Pazdur R (2003) Velcade: U.S. FDA approval for the treatment of multiple myeloma progressing on prior therapy. Oncologist 8, 508–513. 14657528 10.1634/theoncologist.8-6-508

[mol212641-bib-0020] Kang R , Zeh HJ , Lotze MT and Tang D (2011) The Beclin1 network regulates autophagy and apoptosis. Cell Death Differ 18, 571–580. 21311563 10.1038/cdd.2010.191 PMC3131912

[mol212641-bib-0021] Kocaturk NM , Akkoc Y , Kig C , Bayraktar O , Gozuacik D and Kutlu O (2019) Autophagy as a molecular target for cancer treatment. Eur J Pharm Sci 134, 116–137. 30981885 10.1016/j.ejps.2019.04.011

[mol212641-bib-0022] Kokuryo T , Senga T , Yokoyama Y , Nagino M , Nimura Y and Hamaguchi M (2007) Nek2 as an effective target for inhibition of tumorigenic growth and peritoneal dissemination of cholangiocarcinoma. Cancer Res 67, 9637–9642. 17942892 10.1158/0008-5472.CAN-07-1489

[mol212641-bib-0023] Liu J , Xia H , Kim M , Xu L , Li Y , Zhang L , Cai Y , Norberg HV , Zhang T , Furuya T *et al* (2011) Beclin1 controls the levels of p53 by regulating the deubiquitination activity of USP10 and USP13. Cell 147, 223–234. 21962518 10.1016/j.cell.2011.08.037 PMC3441147

[mol212641-bib-0024] Lu Y , Wang Y , Xu H , Shi C , Jin F and Li W (2018) Profilin 1 induces drug resistance through Beclin1 complex‐mediated autophagy in multiple myeloma. Cancer Sci 109, 2706–2716. 29945297 10.1111/cas.13711 PMC6125445

[mol212641-bib-0025] Menon MB and Dhamija S (2018) Beclin 1 phosphorylation at the center of autophagy regulation. Front Cell Dev Biol 6, 9. 30370269 10.3389/fcell.2018.00137 PMC6194997

[mol212641-bib-0026] Mizushima N and Komatsu M (2011) Autophagy: renovation of cells and tissues. Cell 147, 728–741. 22078875 10.1016/j.cell.2011.10.026

[mol212641-bib-0027] Navé BT , Ouwens M , Withers DJ , Alessi DR and Shepherd PR (1999) Mammalian target of rapamycin is a direct target for protein kinase B: identification of a convergence point for opposing effects of insulin and amino‐acid deficiency on protein translation. Biochem J 344, 427–431. 10567225 PMC1220660

[mol212641-bib-0028] Neal CP , Fry AM , Moreman C , McGregor A , Garcea G , Berry DP and Manson MM (2014) Overexpression of the Nek2 kinase in colorectal cancer correlates with beta‐catenin relocalization and shortened cancer‐specific survival. J Surg Oncol 110, 828–838. 25043295 10.1002/jso.23717

[mol212641-bib-0029] Ohashi Y , Tremel S and Williams RL (2019) VPS34 complexes from a structural perspective. J Lipid Res 60, 229–241. 30397185 10.1194/jlr.R089490 PMC6358306

[mol212641-bib-0030] Palumbo A and Anderson K (2011) Multiple myeloma. N Engl J Med 364, 1046–1060. 21410373 10.1056/NEJMra1011442

[mol212641-bib-0031] Roy M , Liang L , Xiao X , Peng Y , Luo Y , Zhou W , Zhang J , Qiu L , Zhang S , Liu F *et al* (2016) Lycorine downregulates HMGB1 to inhibit autophagy and enhances bortezomib activity in multiple myeloma. Theranostics. 6, 2209–2224. 27924158 10.7150/thno.15584 PMC5135444

[mol212641-bib-0032] Sirohi B and Ray P (2004) Multiple myeloma. Lancet 363, 875–887. 15031034 10.1016/S0140-6736(04)15736-X

[mol212641-bib-0033] Song MS , Salmena L , Carracedo A , Egia A , Lo‐Coco F , Teruya‐Feldstein J and Pandolfi PP (2008) The deubiquitinylation and localization of PTEN are regulated by a HAUSP‐PML network. Nature 455, 813–819. 18716620 10.1038/nature07290 PMC3398484

[mol212641-bib-0034] Suzuki K , Kokuryo T , Senga T , Yokoyama Y , Nagino M and Hamaguchi M (2010) Novel combination treatment for colorectal cancer using Nek2 siRNA and cisplatin. Cancer Sci 101, 1163–1169. 20345485 10.1111/j.1349-7006.2010.01504.x PMC11159639

[mol212641-bib-0035] Toton E , Lisiak N , Sawicka P and Rybczynska M (2014) Beclin‐1 and its role as a target for anticancer therapy. J Physiol Pharmacol 65, 459–467. 25179078

[mol212641-bib-0036] Tsunoda N , Kokuryo T , Oda K , Senga T , Yokoyama Y , Nagino M , Nimura Y and Hamaguchi M (2009) Nek2 as a novel molecular target for the treatment of breast carcinoma. Cancer Sci 100, 111–116. 19038001 10.1111/j.1349-7006.2008.01007.x PMC11158353

[mol212641-bib-0037] Vogl DT , Stadtmauer EA , Tan KS , Heitjan DF , Davis LE , Pontiggia L , Rangwala R , Piao S , Chang YC , Scott EC *et al* (2014) Combined autophagy and proteasome inhibition: a phase 1 trial of hydroxychloroquine and bortezomib in patients with relapsed/refractory myeloma. Autophagy 10, 1380–1390. 24991834 10.4161/auto.29264 PMC4203515

[mol212641-bib-0038] Wang S , Li W , Liu N , Zhang F , Liu H , Liu F , Liu J , Zhang T and Niu Y (2012) Nek2A contributes to tumorigenic growth and possibly functions as potential therapeutic target for human breast cancer. J Cell Biochem 113, 1904–1914. 22234886 10.1002/jcb.24059

[mol212641-bib-0039] Xia J , Franqui‐Machin R , Gu Z and Zhan F (2015) Role of NEK2A in human cancer and its therapeutic potentials. Biomed Res Int 2015, 12. 10.1155/2015/862461 PMC4330945 25705694

[mol212641-bib-0040] Xia J , Xu H , Zhang X , Allamargot C , Coleman KL , Nessler R , Frech I , Tricot G and Zhan F (2017) Multiple myeloma tumor cells are selectively killed by pharmacologically‐ dosed ascorbic acid. EBioMedicine 18, 41–49. 28229908 10.1016/j.ebiom.2017.02.011 PMC5405162

[mol212641-bib-0041] Yang Y , Zhou W , Xia J , Gu Z , Wendlandt E , Zhan X , Janz S , Tricot G and Zhan F (2014) NEK2 mediates ALDH1A1‐dependent drug resistance in multiple myeloma. Oncotarget 5, 11986–11997. 25230277 10.18632/oncotarget.2388 PMC4322982

[mol212641-bib-0042] Zhang H , Pang Y , Ma C , Li J , Wang H and Shao Z (2018) ClC5 decreases the sensitivity of multiple myeloma cells to bortezomib via promoting prosurvival autophagy. Oncol Res 26, 421–429. 28899456 10.3727/096504017X15049221237147 PMC7844740

[mol212641-bib-0043] Zhang M , He J , Liu Z , Lu Y , Zheng Y , Li H , Xu J , Liu H , Qian J , Orlowski RZ *et al* (2015) Anti‐β2‐microglobulin monoclonal antibodies overcome bortezomib resistance in multiple myeloma by inhibiting autophagy. Oncotarget 6, 8567–8578. 25895124 10.18632/oncotarget.3251 PMC4496167

[mol212641-bib-0044] Zhang X , Li W , Wang C , Leng X , Lian S , Feng J , Li J and Wang H (2014) Inhibition of autophagy enhances apoptosis induced by proteasome inhibitor bortezomib in human glioblastoma U87 and U251 cells. Cancer Lett 385, 265–275. 10.1007/s11010-013-1835-z PMC3840293 24104452

[mol212641-bib-0045] Zhou W , Yang Y , Xia J , Wang H , Salama ME , Xiong W , Xu H , Shetty S , Chen T , Zeng Z *et al* (2013) NEK2 induces drug resistance mainly through activation of efflux drug pumps and is associated with poor prognosis in myeloma and other cancers. Cancer Cell 23, 48–62. 23328480 10.1016/j.ccr.2012.12.001 PMC3954609

