# Supplementary material for: NEK2 induces autophagy‐mediated bortezomib resistance by stabilizing Beclin‐1 in multiple myeloma
Source: Mol Oncol. 2020 Jan 29;14(4):763–78. doi: 10.1002/1878-0261.12641 (PMC7138399; doi:10.1002/1878-0261.12641)
Supplement: Supplementary file 1 — Fig. S1. NEK2 regulates Beclin‐1 at protein level but not affects its mRNA expression and phosphorylation. Fig. S2. Beclin‐1 is regulated by proteasome inhibitors. Table S1. Clinical characteristics of healthy donors and MM patients. Table S2. The list of primer sequences. [file MOL2-14-763-s001.pdf]

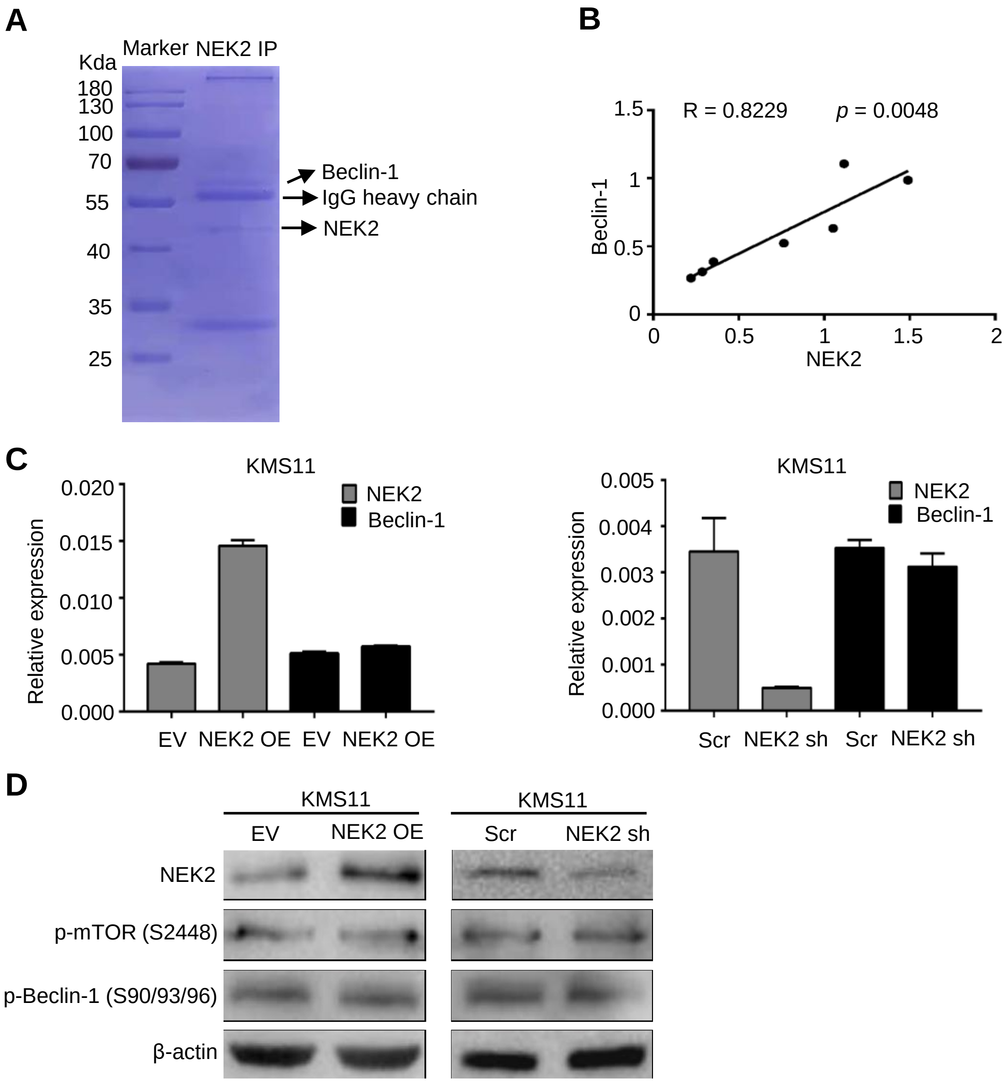

Fig. S1. NEK2 regulates Beclin-1 at protein level but not affects its mRNA expression and phosphorylation. (A) Proteins binding to NEK2 were pulled down by NEK2 antibodies and stained with coomassie brilliant blue (CBB) R250 prior to MS. (B) The expressions of NEK2 and Beclin-1 relative to β-actin were quantified, the expression correlation between NEK2 and Beclin-1 was analyzed by pearson correlation coefficient. (C) Relative mRNA levels of NEK2 and BECN1 in KMS11 EV, KMS11 NEK2 OE, KMS11 Scr, KMS11 NEK2 sh, RPMI 8226 EV, RPMI 8226 NEK2 OE, RPMI 8226 Scr, and RPMI 8226 NEK2 sh. (D) Western blots of NEK2, p-mTOR (S2448), p-Beclin-1 (S90/93/96) and β-actin in KMS11 EV, KMS11 NEK2 OE, KMS11 Scr, and KMS11 NEK2 sh. Error bars indicate SD.

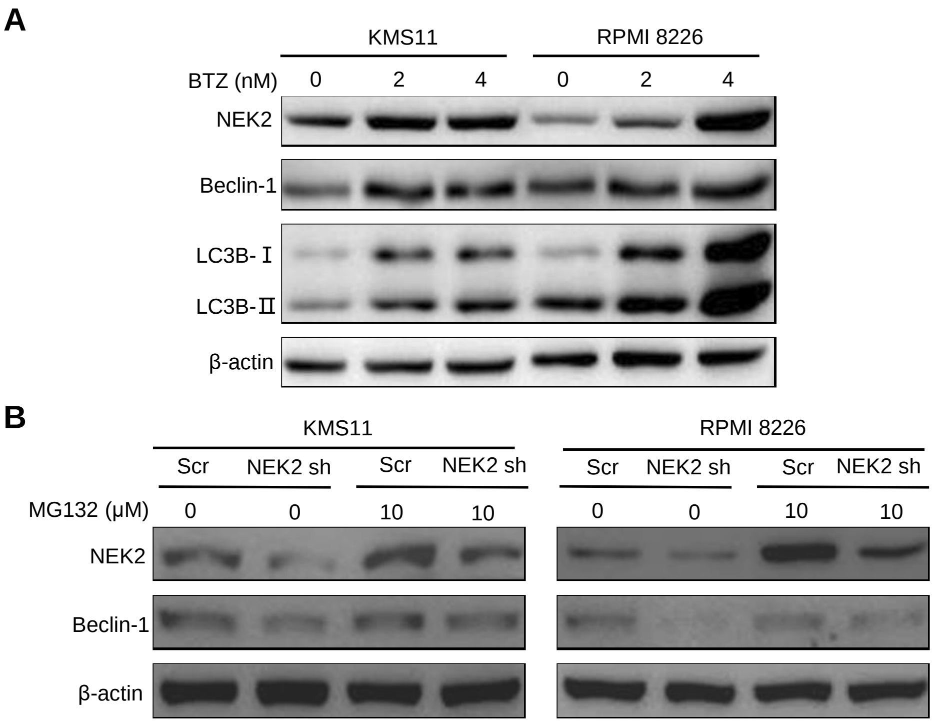

Fig. S2. Beclin-1 is regulated by proteasome inhibitors. (A) KMS11 and RPMI 8226 were treated with BTZ (0, 2 nM, 4 nM) for 24 hours, cells were lysed, and then the levels of NEK2, Beclin-1, LC3B and β-actin were analyzed by western blotting. (B) KMS11 Scr, KMS11 NEK2 sh, RPMI 8226 Scr, and RPMI 8226 NEK2 sh were treated with MG132 (0, 10 μM) for 10 hours, cells were lysed, the levels of NEK2, Beclin-1 and β-actin were analyzed by western blotting.

Table S1. Clinical characteristics of healthy donors and MM patients

Subject number	Disease	Gender	Age	M-component type	Stage (ISS)	Plasma Cell (%)	Cytogenetics	Last treatment
1	HD	60	M					
2	HD	44	M					
3	HD	45	M					
4	HD	50	M					
5	HD	25	F					
6	HD	24	M					
7	NDMM	67	M	IgA Lambda	Ⅲ	12	Normal Fish	
8	NDMM	44	M	IgA Kappa	Ⅲ	1	1q21 amplification	
9	NDMM	49	F	Kappa light chain only	Ⅰ	3.5	Normal Fish	
10	NDMM	56	F	Lambda light chain only	Ⅰ	3.5	Normal Fish	
11	NDMM	76	F	IgM Lambda	Ⅲ	57	Normal Fish	
12	NDMM	70	M	IgA Kappa	Ⅲ	35	Normal Fish	
13	NDMM	66	M	Lambda Light chain only	Ⅱ	68	1q21 amplification, RB deletion	
14	NDMM	48	F	IgG Lambda	Ⅰ	20	RB1 deletion, D13S319 deletion	
15	NDMM	52	F	IgG Kappa	Ⅲ	8.9	Normal Fish	
16	RMM	67	M	IgG Lambda	Ⅲ	3.5	T (11;14) (q13; q32)	VD
17	RMM	49	F	Lambda light chain only	Ⅰ	3.5	Normal Fish	VD
18	RMM	46	M	Kappa light chain only	Ⅰ	15.5	1q21 amplification, RB deletion	VTD
19	RMM	64	M	Kappa light chain only	Ⅲ	5.3	Normal Fish	VCD
20	RMM	48	M	IgA Kappa	Ⅱ	45.5	Normal Fish	VD
21	RMM	66	F	IgG Lambda	Ⅱ	48	1q21 amplification, IgH translocation, 13q deletion, t (14;16) (q32; q23)	VAD
22	RMM	69	F	IgG Lambda	Ⅲ	13.5	Normal Fish	VAD

HD, healthy donors; NDMM, Newly diagnosed MM patients; RMM, Relapsed MM patients; M, Male; F, Female; VD, Bortezomib + Dexamethasone; VAD, Bortezomib + Adriamycin + Dexamethasone; VCD, Bortezomib + Cyclophosphamide + Dexamethasone; VTD, Bortezomib + Thalidomide + Dexamethasone.

Table S2. The list of primer sequences

Primer name	Sequence
NEK2 XbaⅠ sense	TGGTCTAGAGCCACCATGCCTTCCCGGGCTGAG
NEK2 BamHⅠ antisense	TCTGGATCCCTAGCGCATGCCCAGGAT
NEK2 shRNA sense	CCGGCGTTACTCTGATGAATTGAATCTCGAGATTCAATTCATCAGAGTAACGTTTTTG
NEK2 shRNA antisense	AATTCAAAAACGTTACTCTGATGAATTGAATCTCGAGATTCAATTCATCAGAGTAACG
BECN1 shRNA sense	CCGGCCCGTGGAATGGAATGAGATTCTCGAGAATCTCATTCCATTCCACGGGTTTTTG
BECN1 shRNA antisense	AATTCAAAAACCCGTGGAATGGAATGAGATTCTCGAGAATCTCATTCCATTCCACGGG
Scramble shRNA sense	CCGGGCGCGATAGCGCTAATAATTTCTCGAGAAATTATTAGCGCTATCGCGCTTTTT
Scramble shRNA antisense	AATTAAAAAGCGCGATAGCGCTAATAATTTCTCGAGAAATTATTAGCGCTATCGCGC
NEK2 QPCR sense	AGGAATGCCACAGACGAA
NEK2 QPCR antisense	TACAGCAAGCAGCCCAAT
BECN1 QPCR sense	CTGGACACTCAGCTCAACGTCA
BECN1 QPCR antisense	CTCTAGTGCCAGCTCCTTTAGC
β-actin QPCR sense	GTCTTCCCCTCCATCGTG
β-actin QPCR antisense	TTCTCCATGTCGTCCCAG
